# From Nonlinear Optimization to Convex Optimization through Firefly Algorithm and Indirect Approach with Applications to CAD/CAM

**DOI:** 10.1155/2013/283919

**Published:** 2013-11-24

**Authors:** Akemi Gálvez, Andrés Iglesias

**Affiliations:** ^1^Department of Applied Mathematics and Computational Sciences, E.T.S.I. Caminos, Canales y Puertos, University of Cantabria, Avenida de los Castros s/n, 39005 Santander, Spain; ^2^Department of Information Science, Faculty of Sciences, Toho University, 2-2-1 Miyama, Funabashi 274-8510, Japan

## Abstract

Fitting spline curves to data points is a very important issue in many applied fields. It is also challenging, because these curves typically depend on many continuous variables in a highly interrelated nonlinear way. In general, it is not possible to compute these parameters analytically, so the problem is formulated as a continuous nonlinear optimization problem, for which traditional optimization techniques usually fail. This paper presents a new bioinspired method to tackle this issue. In this method, optimization is performed through a combination of two techniques. Firstly, we apply the indirect approach to the knots, in which they are not initially the subject of optimization but precomputed with a coarse approximation scheme. Secondly, a powerful bioinspired metaheuristic technique, the firefly algorithm, is applied to optimization of data parameterization; then, the knot vector is refined by using De Boor's method, thus yielding a better approximation to the optimal knot vector. This scheme converts the original nonlinear continuous optimization problem into a convex optimization problem, solved by singular value decomposition. Our method is applied to some illustrative real-world examples from the CAD/CAM field. Our experimental results show that the proposed scheme can solve the original continuous nonlinear optimization problem very efficiently.

## 1. Introduction

 Fitting spline curves to data points is a problem that appears very frequently in many scientific and engineering fields. Typical examples span from regression analysis in statistics [1, 2] to contour reconstruction in medical imaging [3]. They also encompass the computation of outlines in image processing [4], shape manipulation in geometric modeling and processing [5–7], and data approximation methods in numerical analysis [8, 9], to mention just a few examples. In this paper, our main motivation comes from the fields of computer-aided design and manufacturing (CAD/CAM) where spline curves are intensively used in many problems [10–14]. One of them is to fit data points obtained from metrology in CAD/CAM, a field consisting of the application of measurement technology to the quality control assessment of designed or manufactured products in many manufacturing industries (automotive, aerospace, ship building, shoes, etc.).

In spite of its wide range of applications, the use of spline curves is still challenging because they typically depend on many different continuous variables (data parameters, knots, and spline coefficients) in a highly nonlinear way [15–20]. These sets of variables are also interrelated, meaning that changes in the values of a particular set of parameters affect the behavior of the others, and hence they cannot be manipulated independently [21–24]. For instance, the choice of knots depends on the curve parameterization, which in turn depends on the underlying structure of data points. Similarly, the computation of the spline coefficients depends on both the parameterization and the knots, and so on. From a mathematical standpoint, this implies that the fitting problem cannot be partitioned into independent subproblems for the different sets of variables. As a consequence, it is not possible in general to compute all these parameters analytically [19, 25]. Instead, the typical formulation in the field is to treat this problem as a continuous nonlinear optimization problem [26, 27]. The bottom point is that traditional optimization techniques have also failed to provide satisfactory answer to this optimization problem. Among the alternatives suggested to solve this limitation, those based on artificial intelligence techniques captured the interest of the scientific community some years ago. The main line of research focused on the neural networks [28–30] and their extension, the functional networks [31–34]. However, the solutions reported were partial and applicable only to some particular problems. Consequently, there is a need for more efficient approaches to tackle this issue.

During the last few years, scientists and engineers have turned their attention to *bioinspired computation*, a field where the interplay between nature and computers has allowed us to model the living phenomena by using mathematics and computer science [35–37]. Simultaneously, the study of life has led to improved schemes to solve many problems in mathematics and computer science, including optimization problems [38–40]. Due to their good behavior for complex optimization problems involving ambiguous and noisy data, there has recently been an increasing interest in applying bioinspired optimization techniques to the spline fitting problem. However, there are still few works reported in the literature. Recent schemes in this area are described for particle swarm optimization [41–43], genetic algorithms [27, 44, 45], artificial immune systems [46, 47], estimation of distribution algorithms [48], and hybrid approaches [49–51].

Specially remarkable is the fact that some bioinspired methods have proved to be able to solve difficult optimization problems unsolvable with traditional optimization techniques. Being this our case, we turned our attention to a powerful bioinspired metaheuristic called firefly algorithm, recently introduced by Professor Xin-She Yang to solve difficult continuous optimization problems. The firelfy algorithm is inspired in the flashing behavior of the fireflies and their social interaction in the natural environment (see Section 3 for details).

In this paper, we present a new bioinspired scheme for computing all parameters of a spline curve approximating a given set of data points. Our proposal is based on two fundamental techniques: the indirect approach and the firefly algorithm, which are combined in our method to perform the optimization of the knots and the data parameters, respectively. The indirect approach tries to overcome the fact that computing the knots requires a previous parameterization which, at its turn, requires a previous knot vector, leading in practice to a never-ending vicious circle. In the indirect approach, the knots are not initially the subject of optimization but precomputed with a coarse approximation scheme, which will be further improved at a later stage. This precomputed knot vector plays the role of an initial seed for the data parameterization step. An obvious risk of this indirect approach is that the whole method relies on this optimization stage. In this way, data parameterization becomes the most critical step, since it carries out the most significant part of the optimization effort. Consequently, we need a powerful, reliable optimization method for this task. As it will be shown later on, the firefly algorithm is a good choice for this step. It is applied in the second step to perform optimization on data parameterization; then, the knot vector is refined by using De Boor's method, thus yielding a better approximation of the optimal knot vector. These two combined methods convert the original nonlinear continuous optimization problem into a convex optimization problem, which is solved by applying singular value decomposition. This scheme is applied to some illustrative real-world examples from the CAD/CAM field, including the side profile curve of a car body, the outline curves of a paint spray gun, and a 3D CAD/CAM workpiece from the automotive industry. Our experimental results show that the proposed scheme can solve the original continuous nonlinear optimization problem very efficiently.

The structure of this paper is as follows. Firstly, some basic concepts about parametric spline curves are given in Section 2. Then, Section 3 describes the firefly algorithm, the bioinspired metaheuristic used in this paper. The core of the paper is in Section 4, where our proposed method for spline curve fitting is reported in detail.  Section 5 describes the experimental results of the application of our method to three illustrative real-world problems from the CAD/CAM field. The paper closes with the main conclusions and our plans for future work.

## 2. Parametric Spline Curves

In this section we describe the basic concepts needed in this paper about the parametric spline functions. The interested reader is referred to [6, 7, 52, 53] for a more detailed discussion about this subject. Note that in this paper vectors are denoted in bold.

Let Φ(*τ*) = (*ϕ* ^1^(*τ*),…, *ϕ* ^*n*^(*τ*)) be a parametric function defined on a finite interval [*α*, *β*]. Consider now a strictly increasing sequence of real numbers *μ*_0_ = *α* < *μ*_1_ < ⋯*μ*_*υ*_ < *μ*_*υ*+1_ = *β* called knots. The function Φ(*τ*) is a parametric polynomial spline of degree *η* ≥ 0 with knots {*μ*_*k*_}_*k*_ if the following two conditions are fulfilled for *i* = 0,…, *υ* and *j* = 1,…, *n*:

1. *ϕ* ^*j*^(*τ*) is a polynomial spline of degree up to *η* on each interval [*μ*_*i*_, *μ*_*i*+1_],
2. *ϕ* ^*j*^(*τ*) and its derivatives up to order *η* − 1 are continuous on [*μ*_*i*_, *μ*_*i*+1_].

Different basis functions can be used for polynomial splines. In this paper, we consider the B-spline basis functions of degree *ν* defined on [*μ*_*i*_, *μ*_*i*+1_] according to the Cox-de-Boor recursive formula [52]:

(1) ψi,ν+1(τ)=φi,ν+(τ)ψi,ν(τ)+φi+1,ν−(τ)ψi+1,ν(τ),              i=0,…,υ−ν, ν>1,

where *φ*_*i*,*ν*_^+^(*τ*) = (*τ* − *μ*_*i*_)/(*μ*_*i*+*ν*_ − *μ*_*i*_), *φ*_*i*,*ν*_^−^(*τ*) = (*μ*_*i*+*ν*_ − *τ*)/(*μ*_*i*+*ν*_ − *μ*_*i*_), and *ψ*_*i*,1_(*τ*) is the unit function with support on the interval [*μ*_*i*_, *μ*_*i*+1_). The dimension of the vector space of functions satisfying conditions (1) and (2) is *υ* + *η* + 1. The given knot vector {*μ*_*i*_}_*i*_ yields *υ* − *η* + 1 linearly independent basis functions of degree *η*. The remaining 2*η* basis functions are obtained by introducing the boundary knots *μ*_−*η*_ = *μ*_−*η*+1_ = ⋯ = *μ*_−1_ = *μ*_0_ = *α* and *μ*_*υ*+1_ = *μ*_*υ*+2_ = ⋯ = *μ*_*υ*+*η*+1_ = *β*. With this choice of boundary knots all basis functions vanish outside the interval domain [*α*, *β*]. Every parametric spline curve Φ(*τ*) is represented by

(2) $$\begin{array}{c} \Phi \left(\tau \right)=\sum_{i=-\eta }^{\upsilon }\Xi_{i}\;\psi_{i,\eta +1}\left(\tau \right), \end{array}$$

where {**Ξ**_*i*_} are the spline coefficients of the curve and *ψ*_*i*,*η*+1_(*τ*) are the basis functions defined above. The *k*th derivative of Φ(*τ*) is a spline of degree *η* − *k* given by

(3) $$\begin{array}{c} \Phi^{\left(k\right)}\left(\tau \right)=\prod_{i=1}^{k}\left(\eta +1-i\right)\sum_{i=-\eta +k}^{\upsilon }\Xi_{i}^{\left(k\right)}\psi_{i,\eta +1-k}\left(\tau \right) \end{array}$$

with **Ξ**_*i*_^(*j*)^ = (**Ξ**_*i*_^(*j*−1)^ − **Ξ**_*i*−1_^(*j*−1)^)/(*μ*_*i*+*η*+1−*j*_ − *μ*_*i*_) for *j* > 0 and **Ξ**_*i*_^(0)^ = **Ξ**_*i*_.

## 3. The Firefly Algorithm

 The firefly algorithm (FFA) is a bioinspired metaheuristic algorithm introduced in 2008 by Yang to solve optimization problems [54–58]. The algorithm is based on the flashing behavior of the fireflies and their social interaction in the natural environment. The key ingredients of the method are the variation of light intensity and formulation of attractiveness. In general, the attractiveness of an individual is assumed to be proportional to their brightness, which in turn is associated with the encoded objective function. The reader is kindly referred to [40] for a comprehensive review of the firefly algorithm and other nature-inspired metaheuristic approaches. See also [59] for a gentle introduction to metaheuristic applications in engineering optimization.

In the firefly algorithm, there are three particular idealized rules, which are based on some of the major flashing characteristics of real fireflies [54] as follows

1. All fireflies are unisex, so that one firefly will be attracted to other fireflies regardless of their sex.
2. The degree of attractiveness of a firefly is proportional to its brightness, which decreases as the distance from the other firefly increases due to the fact that the air absorbs light. For any two flashing fireflies, the less bright one will move towards the brighter one. If there is not a brighter or more attractive firefly than a particular one, it will then move randomly.
3. The brightness or light intensity of a firefly is determined by the value of the objective function of a given problem. For instance, for maximization problems, the light intensity can simply be proportional to the value of the objective function.

The distance between any two fireflies *i* and *j*, at positions **P**_*i*_ and **P**_*j*_, respectively, can be defined as a Cartesian or Euclidean distance as follows:

(4) $$\begin{array}{c} r_{ij}={||P_{i}-P_{j}||}_{2}=\sqrt{\sum_{k=1}^{D}{\left(p_{i}^{k}-p_{j}^{k}\right)}^{2}}, \end{array}$$

where *p*_*i*_^*k*^ is the *k*th component of the spatial coordinate **P**_*i*_ of the *i*th firefly and *D* is the number of dimensions. In the firefly algorithm, as attractiveness function of a firefly *j* one should select any monotonically decreasing function of the distance to the chosen firefly, for example, the exponential function:

(5) $$\begin{array}{c} \hat{\beta }={\hat{\beta }}_{0}e^{-\hat{\gamma }r_{ij}^{\hat{\mu }}},\;\hat{\mu }\geq 1, \end{array}$$

where *r*_*ij*_ is the distance defined as in (4), ${\hat{\beta }}_{0}$ is the initial attractiveness at *r* = 0, and $\hat{\gamma }$ is an absorption coefficient at the source which controls the decrease of the light intensity.

The movement of a firefly *i* which is attracted by a more attractive (i.e., brighter) firefly *j* is governed by the following evolution equation:

(6) $$\begin{array}{c} P_{i}^{\left(t+1\right)}=P_{i}^{\left(t\right)}+{\hat{\beta }}_{0}e^{-\hat{\gamma }r_{ij}^{\hat{\mu }}}\left(P_{j}^{\left(t\right)}-P_{i}^{\left(t\right)}\right)+\hat{\alpha }\left(\hat{\sigma }-\frac{1}{2}\right), \end{array}$$

where the superscripts between brackets in this expression are used to denote the corresponding generations. The first term on the right-hand side is the current position of the firefly at generation *t*, the second term is used for considering the attractiveness of the firefly to light intensity seen by adjacent fireflies, and the third term is used for the random movement of a firefly in case there are not any brighter ones. The coefficient $\hat{\alpha }$ is a randomization parameter determined by the problem of interest, while $\hat{\sigma }$ is a random number generator uniformly distributed in the space [0,1].

The method described in previous paragraphs corresponds to the original version of the firefly algorithm, as originally developed by its inventor. Since then, many different modifications and improvements on the original version have been developed, including the discrete FFA, multiobjective FFA, chaotic FFA, parallel FFA, elitist FFA, Lagrangian FFA, and many others, including its hybridization with other techniques. The interested reader is referred to the nice paper in [60] for a comprehensive, updated review and taxonomic classification of the firefly algorithms and all its variants and applications.

## 4. The Method

 In this section our FFA-based method is fully explained. The section begins with the description of the optimization problem to be solved. Then, a general overview of the method and its flowchart are given. Then, each step of the method is discussed in detail. Finally, some details regarding the implementation issues are also given.

### 4.1. The Optimization Problem

 Let us suppose that we are provided with a set of measured data points {Θ_*k*_}_*k*=1,…,*ρ*_ ⊂ ℝ^*n*^ obtained by laser scanning, layout machine, or other digitizing methods, as it typically happens in many scientific and engineering problems. The goal consists of obtaining a parametric spline curve Φ(*τ*) of degree *η* defined as above approximating the {Θ_*k*_}_*k*_. Due to the conditions on the boundary knots, we can take Φ(*τ*_1_) = Θ_1_ and Φ(*τ*_*ρ*_) = Θ_*ρ*_ and perform approximation on the remaining parameters; that is,

(7) $$\begin{array}{c} \Theta_{k}\approx \Phi \left(\tau_{k}\right)=\sum_{j=-\eta }^{\upsilon }\Xi_{j}\psi_{j,\eta +1}\left(\tau_{k}\right)\;\left(k=2,\ldots ,\rho -1\right). \end{array}$$

Equation (7) can be written in matrix notation as

(8) $$\begin{array}{c} \Theta =\Psi \cdot \Xi , \end{array}$$

where Θ = (Θ_2_,…, Θ_*ρ*−1_)^*T*^, **Ξ** = (**Ξ**_−*η*_,…, **Ξ**_*υ*_)^*T*^, Ψ = ({*ψ*_*j*,*η*+1_(*τ*_*k*_)}_*j*=2,…,*ρ*−1;  *k*=−*η*,…,*υ*_) is the matrix of sampled spline basis functions, and (·)^*T*^ represents the transpose of a vector or matrix. The dimension of the search space *D* in (8) is given by *n*(*υ* + *η* − 1) + *υ* + *ρ* − 2, which could be of several thousands of variables for nontrivial shapes. Since the system (8) is overdetermined, the matrix of basis functions is not invertible and no direct solution can be obtained. Therefore, we consider the least-squares approximation of (7), defined as the minimization problem given by

(9) $$\begin{array}{c} \underset{\overset{{\left\{\mu_{i}\right\}}_{i}}{\overset{{\left\{\Xi_{j}\right\}}_{j}}{{\left\{\tau_{k}\right\}}_{k}}}}{\text{minimize}}\left(\sum_{k=2}^{\rho -1}\omega_{k}{||\Theta_{k}-\sum_{j=-\eta }^{\upsilon }\Xi_{j}\psi_{j,\eta +1}\left(t_{k}\right)||}_{\ell_{2}}\right), \end{array}$$

where *ω*_*k*_ are scalar weights and *ℓ*_2_ represents the Euclidean norm (although any other norm might be used instead). Note that the parameters and knots are related by nonlinear basis functions, thus leading to a high-dimensional continuous nonlinear optimization problem. Assuming that a suitable data parameterization can be obtained, we have to solve a nonlinear continuous optimization problem involving both the spline coefficients and the knots as free variables of the problem. Unfortunately, this approach makes the optimization problem nonconvex, because Φ(*τ*) is a nonconvex function of the knots [19, 26, 52]. To overcome this problem, we follow the so-called indirect approach, in which the knots are precomputed before the optimization process is executed and then refined for better fitting. With this strategy, the resulting problem is convex, so a global optimum can eventually be found. In order to apply the previous strategy, we need to obtain a suitable parameterization of data points, which thus becomes the most critical step of this approach. We solve this parameterization problem by applying the firefly algorithm, as it will be explained in next paragraphs.

### 4.2. Overview of the Method

**Figure 1:**
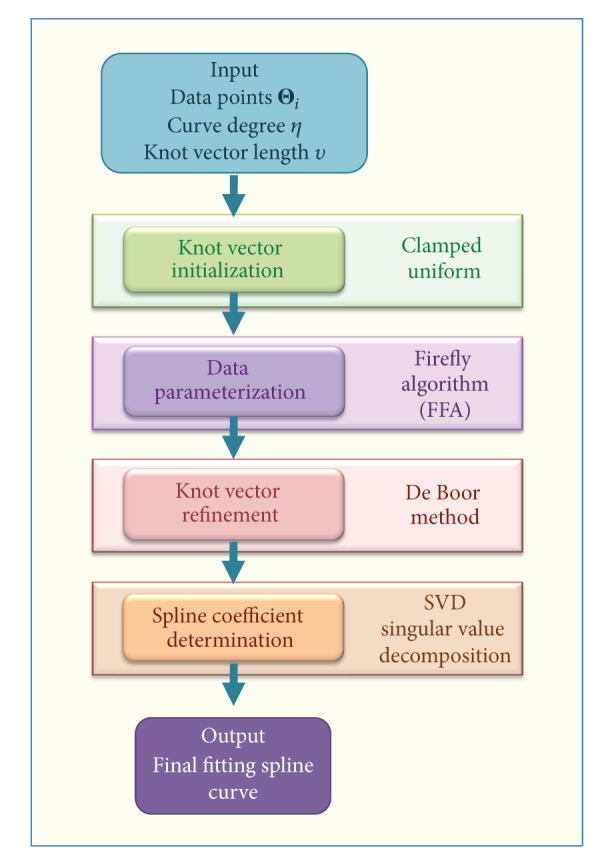
Graphical flowchart of the proposed method.

 The main steps of our method are summarized in Figure 1, showing the flowchart of our approach. Our initial input is given by the set of data points {Θ_*j*_}_*j*_ and two parameters that are freely chosen by the user: the length of knot vector (determined by variable *υ*), and the curve degree *η* (typical values for *η* are between 2 and 4, although any natural value can be used in our method). The first step of our approach consists of computing an initial knot vector. Then, we apply the firefly algorithm to perform data parameterization (Step 2). A new (refined) knot vector is computed based on the parameterization obtained in the previous step. Then, the convex optimization problem is solved by using singular value decomposition (Step 4). After this step, the best fitting spline curve to the data points for the given degree is finally obtained.

### 4.3. Main Steps of the Method

 The proposed method consists of four main steps, analyzed in the next paragraphs.

**Step 1 (knot vector initialization)**

The first step of the method computes an initial knot vector, which is required in order to evaluate the fitness functions during the optimization step for data parameterization. To this aim, we consider a clamped knot vector (this condition is a result of our choice of boundary knots) whereas the internal knots are uniformly distributed on the interval (*α*, *β*); that is, *μ*_*i*_ = *α* + ((*β* − *α*)*i*/(*υ* + 1)), for *i* = 1,…, *υ*. This choice of knots is not optimal because it does not reflect the distribution of data points. This limitation will be overcome in Step 3, where this initial knot vector will be further refined.

**Step 2 (data parameterization)**

In this step, the data points parameterization is carried out. As mentioned above, this is the most critical step of our method; the set of data parameters along with the knot vector, computed in the previous step and refined in the next one, allows us to convert the original nonlinear nonconvex optimization problem (9) into a convex optimization problem. This task is accomplished by applying the firefly algorithm described in Section 3. To this purpose, a collection of *n*_*f*_ particles (fireflies) is considered. Each firefly corresponds to a vector of *ρ* − 2 real numbers on the interval (*α*, *β*). The components of each firefly vector are always sorted in an increasing order to reflect the ordered structure of the data points. The fireflies are initialized by using a random function of uniform distribution on the interval domain (*α*, *β*). We have used a collection of *n*_*f*_ = 100 fireflies for the examples reported in this paper. We also checked our results with larger populations by changing this parameter from 100 to 1000 fireflies with step-size 100 and do not notice significant variations in our results. However, a larger value could be required for very massive sets of data points (≥10^5^ data points) exhibiting very complicated shapes.

Once the initial population of fireflies is generated, some parameters of the firefly algorithm have to be determined in order to apply them to our problem. Our choice of the parameters for the FFA is mostly empirical: we initially rely on standard values reported in the existing literature and then perform computer experiments to validate our parameter tuning. In this paper, we consider the following set of parameter values for the FFA method: ${\hat{\beta }}_{0}=1$, $\hat{\gamma }=0.5$, and $\hat{\mu }=2$. This set of parameter values has already been used in a previous paper by the authors for a Bézier surface parameterization problem with good results [61]. Our computer experiments also confirmed their good performance for the examples discussed in this paper and some others not reported here to keep the paper at manageable size. On the contrary, the number of iterations and the value of parameter $\hat{\alpha }$ required some improvement. We initially used a fixed number of iterations in our experiments as the stopping criterion, but this choice was found to be very inefficient for this problem. The main reasons are that the method can potentially be applied to sets of very different number of data points, meaning that the dimension of the search space can vary dramatically from one example to another, and that the initial knot vector used in our method is not optimal yet, making it difficult to determine in advance how many iterations are needed to achieve convergence. We then turned to a different termination condition, where the number of iterations is determined manually for each specific example, based on the observation of the convergence diagrams (as those shown in Figures 3 and 5). Although it is a tedious and time-consuming task, we found it to be more reliable in order to ensure convergence is properly achieved. Regarding the value for $\hat{\alpha }$, our initial choice $\hat{\alpha }=0.3$ soon revealed to be too drastic, as the fireflies went out of range in just a few iterations (sometimes, even a single one was enough). We decreased its value gradually and carried out a lot of computer simulations. As a result, the best value was found at $\hat{\alpha }=0.01$.

The last required component of the FFA is the fitness function. It corresponds to the evaluation of the least-squares function given by the operator to be minimized in (9); that is,

(10) $$\begin{array}{c} \Omega =\sum_{k=2}^{\rho -1}\omega_{k}{||\Theta_{k}-\sum_{j=-\eta }^{\upsilon }\Xi_{j}\psi_{j,\eta +1}\left(t_{k}\right)||}_{\ell_{2}}, \end{array}$$

where the weights *w*_*k*_ are scalar numbers to express the degree of confidence of data points (larger weights are assigned to more reliable data). In this paper, we assume a constant confidence value for all data, so we take *w*_*k*_ = 1, *k* = 2,…, *ρ* − 1. After the selection of those parameters and the fitness function, the firefly algorithm is performed iteratively until the termination criterion is reached. To remove the stochastic effects of single executions, 20 independent executions have been carried out for each simulation trial. Then, the firefly with the best (i.e., minimum) fitness value is selected as the best solution to the problem. As a result, the best parameterization vector of data points is obtained.

**Step 3 (knot vector refinement)**

Based on the parameterization obtained in the previous step, the knot vector is subsequently refined for better performance. To this purpose, the placement of knots should reflect the distribution of data parameters {*τ*_*k*_}_*k*_. In this paper, the internal knots are computed by following a procedure firstly proposed by De Boor in [52]. The corresponding algorithm is described in Algorithm 1. It has been proved that this algorithm guarantees that every knot span contains at least one *τ*_*k*_. At its turn, this condition ensures that the matrix Γ = Ψ^*T*^Ψ is positive definite and well conditioned. Furthermore, Γ has a semibandwidth less than *η* − 1 (see [52] for details). All these properties imply that the system of equations related to the convex problem can be solved by using efficient and reliable numerical methods [62], as explained in the next paragraphs.

**Step 4 (spline coefficient determination)**

As we mentioned above, in this paper, we follow an indirect approach to compute the fitting spline curve to the data points. In this scheme, data parameters and knots are computed at earlier stages of the optimization process so that the approximation problem (9) becomes a convex problem. In that case, premultiplying by Ψ^*T*^ at both sides of (8), we get

(11) $$\begin{array}{c} \Gamma \cdot \Xi =\Sigma , \end{array}$$

where Σ = Ψ^*T*^ · Θ = ({∑_*k*=2_^*ρ*−1^Θ_*k*_ *ψ*_*i*,*η*+1_(*τ*_*k*_)}_*i*=−*η*,…,*υ*_)^*T*^. Note that Γ = Ψ^*T*^Ψ is a symmetric square matrix and positive semidefinite, so system (11) always has a solution. It can be solved numerically by Gaussian elimination, LU decomposition, or the singular value decomposition (SVD) (see [62] for details). In this paper, SVD has been used since it provides the best numerical answer in the sense of least squares for those cases in which the exact solution is not possible. To this aim, Γ is decomposed as the matrix product Γ = **U** · **W** · **V** ^*T*^, where **U** is a column orthogonal matrix, **W** is a diagonal matrix with positive or zero elements *w*_*k*_ called the singular values, and **V** is a square orthogonal matrix. Furthermore, its inverse can readily be obtained as: Γ^−1^ = **V** · [diag⁡(1/*w*_*k*_)] · **U** ^*T*^. In addition, the knot vector obtained by the procedure described in Step 3 guarantees that Γ is positive, definite, and, therefore, nonsingular, so the problem can be solved by using this inverse matrix Γ^−1^.

### 4.4. Implementation Issues

 Regarding the implementation, all computations in this paper have been performed on a 2.6 GHz Intel Core i7 processor with 8 GB of RAM. The source code has been implemented by the authors in the native programming language of the popular scientific program *Matlab*, version 2012a. In our opinion, *Matlab* is a very suitable tool for this task: it is fast and provides reliable, well-tested routines for efficient matrix manipulations. It also contains a bulk of resources regarding the solving of systems of equations. For instance, *Matlab* provides us with the command mldivide to solve the equation **A** · **X** = **B** for both squared and nonsquared systems (by using Gaussian elimination with partial pivoting and least-squares techniques, resp.). Depending on the general structure of matrix **A**, this command applies specialized LAPACK and BLAS routines to get the best possible solution to this system. Also, *Matlab* provides us with a specialized command svd for computing the SVD of a matrix. This command carries out the matrix SVD decomposition automatically so it can be used in a rather black box-like way. Besides, *Matlab* provides excellent graphical options and optimized code for input/output interaction and high performance computations.

## 5. Experimental Results

 Our method has been applied to several real-world examples of CAD/CAM shapes for the automotive industry. In this section we discuss three of them. The reported examples reflect the variety of cases our method can be applied to: they include both open and closed curves, as well as 2D and 3D shapes comprised of a single curve and multiple curves. We think we have provided enough examples to convince the reader of the broad applicability of our approach.

### 5.1. Side Profile Curve of a Car Body

 The first example consists of the data fitting of a set of 695 data points from the *In *(+*y*-axis) side profile section of a model of a notchback three-box sedan car body. The data points were obtained by a layout machine from the Spanish car maker provider Candemat years ago. This example has been primarily chosen because it includes areas of varying slope, ranging from the strong slope at both ends (upwards on the left, downwards on the right) to the soft slope in middle part, with a horizontal area between pillars A and C and in the cargo box area and a soft upward area in the car hood. In addition, it is a good example of a truly parametric curve that cannot be faithfully represented by simpler functions such as explicit functions and the like. Consequently, it is a very good candidate to check the performance of our approach.

The problem is also challenging because we are trying to represent the whole shape with a single curve. It is worthwhile mentioning here that the use of a single curve for a whole object is not common at all in industrial environments, where the shapes of final products such as a car body are usually represented by a very large set (several hundreds of thousands, even millions) of simpler curves. Therefore, even though we are using data from a real-world shape, this example must be understood as a purely academic example rather than a genuine real-world problem in the automotive industry. Yet, this example is very useful in this paper to analyze the performance of our approach.

**Figure 2:**
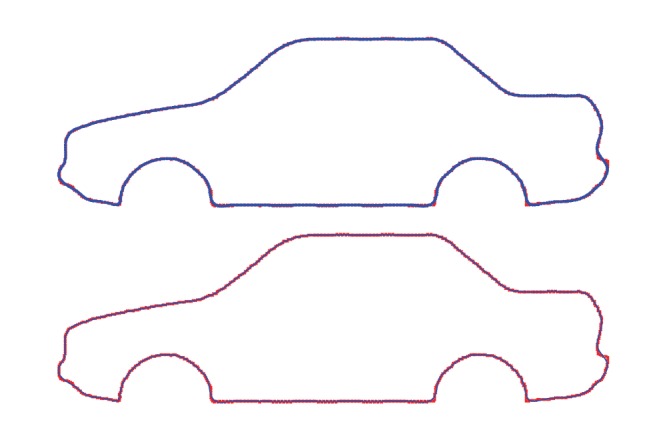
Adjusting data points (red cross symbols in both pictures) of a car body side profile with our method: (top) reconstructed data points (blue empty circle symbols); (bottom) approximating spline curve (blue solid line).

**Figure 3:**
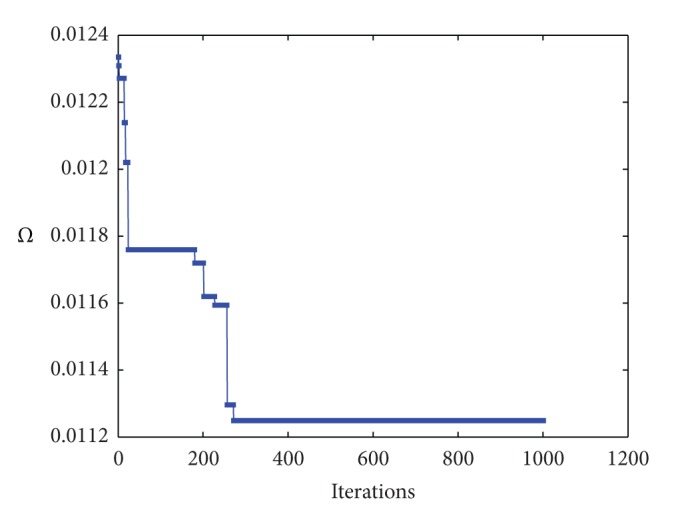
Fitting error evolution of the *Ω* operator for the car body example for 1000 iterations.

Figure 2 shows our simulation results for a spline curve of degree *η* = 3. Top figure shows the original data points, represented by red cross symbols along with the reconstructed data points, displayed as empty circles in blue. The picture is intended to show the correspondence between the original and the reconstructed data points in a graphical way. As the reader can see, our method yields a very good matching between both sets of points. This observation is confirmed by Figure 2 (bottom), representing the same original data points along with the approximating spline curve, displayed as a solid line in blue.  Figure 3 shows the evolution of the fitting error of the *Ω* operator for 1000 iterations. Since the diagram shows a similar general behavior for the 20 independent executions and larger number of iterations no longer improves the fitting error, we conclude that this number of iterations is enough for convergence. The corresponding fitting error in this example is 1.1248 × 10^−2^. This value gives only partial information because it does not consider the number of sampled points. This means that increasing the number of data points leads automatically to larger fitting errors even though each data point might be better fitted. To overcome this drawback, we also compute the root-mean square error (RMSE), which gives a better measure of the quality of the approximation. In this example we obtain an RMSE fitting error of 4.2669 × 10^−4^. This value confirms the good performance of the proposed method for this problem. A close inspection of Figure 2 reveals, however, that some parts can still be further improved: this fact is visually noticeable in Figure 2, specially at the corners of the front and rear fenders and their linkings to the lower car body line, as well as at the lower parts of front and rear bumpers. This effect is attributable to our indirect approach, in which we do not compute the optimal knot vector but an approximation. We are currently working towards an improved approach to solve this limitation.

### 5.2. Outline Curves of a Paint Spray Gun

**Figure 4:**
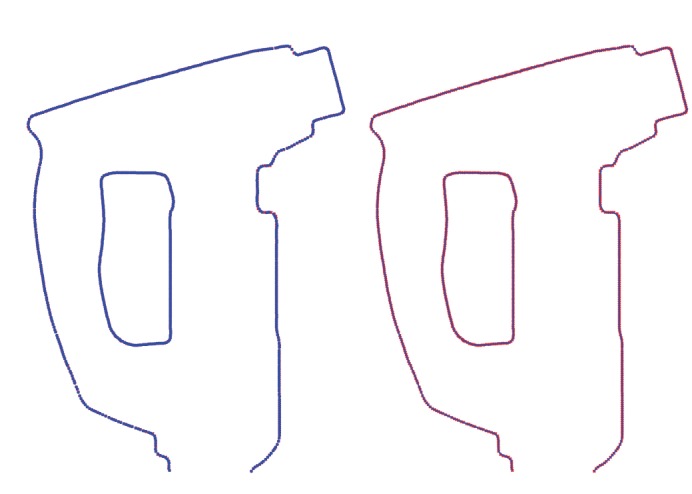
Adjusting data points (red cross symbols in both pictures) of two outlines curves of a paint spray gun with our method: (left) reconstructed data points (blue empty circle symbols); (right) approximating spline curve (blue solid line).

**Figure 5:**
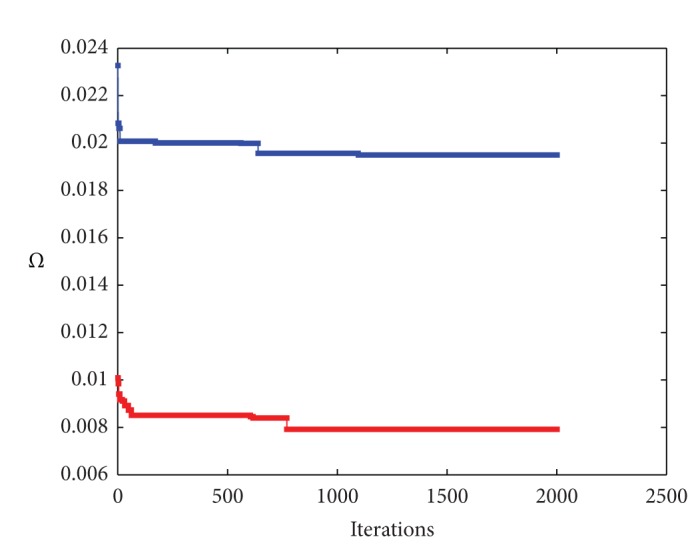
Fitting error evolution of the *Ω* operator for the outer curve (in blue) and the inner curve (in red) of the paint spray gun example for 2000 iterations.

Figure 4 shows the results of our method for spline curves of degree *η* = 3 when applied to a paint spray gun model. Left and right pictures of the figure have the same meaning as the top and bottom pictures of the previous figure, respectively. The spray gun model consists of two different curves for the outer and inner boundary lines with 542 and 276 data points, respectively. We applied our method to each curve independently. The fitting errors of the *Ω* operator for the outer and inner curves are 1.9493 × 10^−2^ and 7.9145 × 10^−3^, respectively. The RMSE fitting errors are 8.3729 × 10^−4^ and 4.7639 × 10^−4^, respectively. These values are reached for 2000 iterations, well within the convergence area as shown in Figure 5, which displays the evolution of the fitting error evolution of the *Ω* operator for 2000 iterations. Fitting errors for the outer and the inner curve in that figure are displayed in blue and red, respectively. Once again, the numerical errors and the visual appearance confirm the good performance of the method in these two cases as well. This example also shows that our approach has a great flexibility, being able to deal with both open and closed curves such as the outer and inner curves of this model, respectively. Note also that even though the data points of the outer curve exhibit many changes of concavity, the method can approximate them very accurately with a single spline curve.

### 5.3. 3D CAD/CAM Workpiece

**Figure 6:**
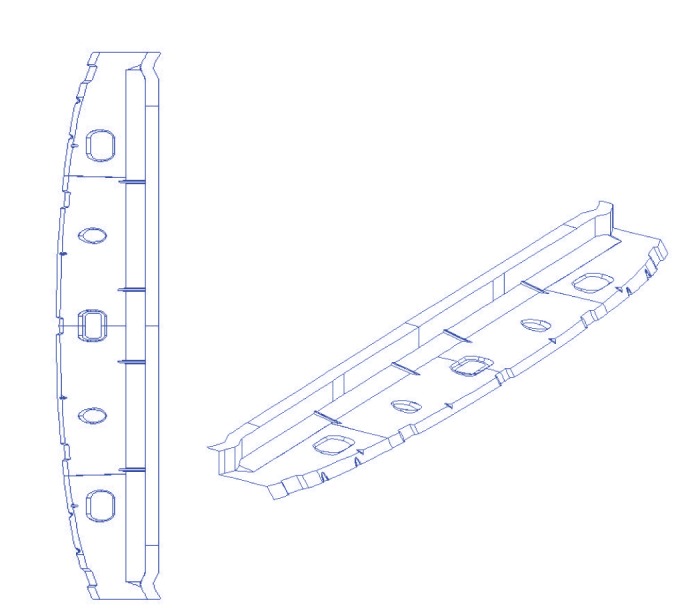
Two different views of a 3D CAD/CAM workpiece comprised of 1610 curves (*courtesy of Candemat*).

Figure 6 illustrates the application of our method to a complex CAD/CAM workpiece of a car body. This example aims at showing the ability of our method to perform well in a real industrial problem involving several geometric shapes. The figure shows two different views of a 3D automotive part comprised of 1610 curves stored in an industrial IGES file obtained from Candemat. As the reader can see, the shape consists of curves with very different topologies, ranging from simple regular shapes such as straight lines and conics to complicated irregular shapes. We therefore applied two different strategies for this example: simple regular shapes are reconstructed by using basic primitives (lines, circles, etc.) for which only some parameters have to be computed (e.g., using two data points for straight lines and three non-aligned data points for the center and radius of a circle), while the complicated shapes are reconstructed with our approach. A total of 243 curves have been reconstructed with our method in Figure 6 by using quadratic and cubic spline curves. The maximum, minimum, and average values of the RMSE fitting error are 3.1729 × 10^−3^, 6.2547 × 10^−4^, and 3.5842 × 10^−6^, respectively. These error values confirm the good performance of our approach for a 3D real-world automotive part comprised of several (both open and closed) spline curves of different degrees.

## 6. Conclusions and Future Work

 In this paper we introduce a new bioinspired method for computing a spline curve that approximates a given set of data points. This task involves many different variables which are interrelated with each other in a nonlinear way, leading to a continuous nonlinear optimization problem. Our approach solves this problem by combining two different procedures for the knots and the data parameters. For the former, an indirect approach that precomputes the knots instead of optimizing them is applied. This strategy leaves the most significant part of the optimization effort to the data parameterization. In our scheme, this task is addressed by a powerful bioinspired metaheuristic technique well suited for difficult continuous optimization problems, the firefly algorithm. Then, the knot vector is refined by using De Boor's method, thus yielding a better approximation to the optimal knot vector. The combination of the indirect approach and the firefly algorithm converts the original nonlinear continuous optimization problem into a convex optimization problem, solved by SVD. The proposed method has been applied to some illustrative real-world examples from the CAD/CAM field involving 2D and 3D open and closed curves. Our experimental results show that the proposed scheme can solve the original continuous nonlinear optimization problem very efficiently.

Regarding the future work, this method can be improved in several ways. As mentioned in Section 5, the indirect approach used in this paper implies that the knot vector is generally very good but not optimal, opening the door for further improvement. We think that a direct approach could lead to better results for the knot vector and, hence, for the overall method. Another interesting field of research is the use of some powerful modifications of the firefly algorithm which have been reported to return better results [63] or extend the capabilities of the standard version for continuous multiobjective optimization [64]. Also, the extension of this approach to other recently described bioinspired methods, such as the cuckoo search [65, 66] or the bat algorithm [67–69], might lead to further improvement of our results. They are all part of our plans for future work in the field.

## Figures and Tables

**Algorithm 1:**
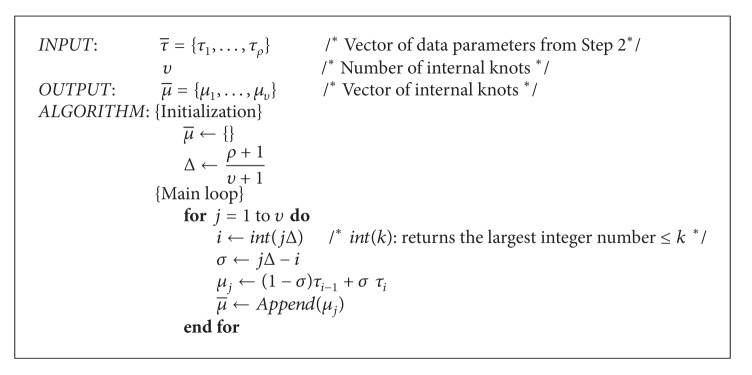
De Boor's knot vector refinement algorithm.

## References

[1] Draper NR, Smith H (1998). *Applied Regression Analysis*.

[2] Molinari N, Durand J-F, Sabatier R (2004). Bounded optimal knots for regression splines. *Computational Statistics and Data Analysis*.

[3] Jacobson TJ, Murphy MJ (2011). Optimized knot placement for B-splines in deformable image registration. *Medical Physics*.

[4] Gonzalez RC, Woods RE (2007). *Digital Image Processing*.

[5] Barnhill RE (1992). *Geometric Processing for Design and Manufacturing*.

[6] Farin G (2002). *Curves and Surfaces for CAGD*.

[7] Hoschek J, Lasser D (1993). *Fundamentals of Computer Aided Geometric Design*.

[8] Dahlquist G, Bjorck A (1974). *Numerical Methods*.

[9] Rice JR (1969). *The Approximation of Functions*.

[10] Alhanaty M, Bercovier M (2001). Curve and surface fitting and design by optimal control methods. *CAD Computer Aided Design*.

[11] Patrikalakis NM, Maekawa T (2002). *Shape Interrogation for Computer Aided Design and Manufacturing*.

[12] Pottmann H, Leopoldseder S, Hofer M, Steiner T, Wang W (2005). Industrial geometry: recent advances and applications in CAD. *CAD Computer Aided Design*.

[13] Várady T, Martin RR, Cox J (1997). Reverse engineering of geometric models—an introduction. *CAD Computer Aided Design*.

[14] Varady T, Martin RR, Farin G, Hoschek J, Kim M (2002). Reverse engineering. *Handbook of Computer Aided Geometric Design*.

[15] Burchard HG (1974). Splines (with optimal knots) are better. *Applicable Analysis*.

[16] de Boor CA, Rice JR (1968). *Least Squares Cubic Spline Approximation—I: Fixed Knots*.

[17] de Boor CA, Rice JR (1968). *Least Squares Cubic Spline Approximation—II: Variable Knots*.

[18] Hölzle GE (1983). Knot placement for piecewise polynomial approximation of curves. *Computer-Aided Design*.

[19] Jupp DLB (1978). Approximation to data by splines with free knots. *SIAM Journal of Numerical Analysis*.

[20] Powell MJD, Hayes JG (1970). Curve fitting by splines in one variable. *Numerical Approximation to Functions and Data*.

[21] Goldenthal R, Bercovier M (2004). Spline curve approximation and design by optimal control over the knots. *Computing*.

[22] Li W, Xu S, Zhao G, Goh LP (2005). Adaptive knot placement in B-spline curve approximation. *CAD Computer Aided Design*.

[23] Lyche T, Mørken K (1987). Knot removal for parametric B-spline curves and surfaces. *Computer Aided Geometric Design*.

[24] Ma W, Kruth J (1995). Parameterization of randomly measured points for least squares fitting of B-spline curves and surfaces. *Computer-Aided Design*.

[25] Lyche T, Mørken K (1988). A data-reduction strategy for splines with applications to the approximation of functions and data. *IMA Journal of Numerical Analysis*.

[26] Dierckx P (1993). *Curve and Surface Fitting with Splines*.

[27] Yoshimoto F, Harada T, Yoshimoto Y (2003). Data fitting with a spline using a real-coded genetic algorithm. *CAD Computer Aided Design*.

[28] Gu P, Yan X (1995). Neural network approach to the reconstruction of freeform surfaces for reverse engineering. *Computer-Aided Design*.

[29] Hoffmann M (2005). Numerical control of kohonen neural network for scattered data approximation. *Numerical Algorithms*.

[30] Knopf GK, Kofman J, Dagli CH Free-form surface reconstruction using Bernstein basis function networks.

[31] Castillo E, Iglesias A (1997). Some characterizations of families of surfaces using functional equations. *ACM Transactions on Graphics*.

[32] Echevarría G, Iglesias A, Gálvez A (2002). Extending neural networks for B-spline surface reconstruction. *Lectures Notes in Computer Science*.

[33] Iglesias A, Echevarría G, Gálvez A (2004). Functional networks for B-spline surface reconstruction. *Future Generation Computer Systems*.

[34] Iglesias A, Gálvez A (2001). A new artificial intelligence paradigm for computer aided geometric design. *Lectures Notes in Artificial Intelligence*.

[35] de Castro LN, Von Zuben FJ (2005). *Recent Developments in Biologically Inspired Computing*.

[36] Floreano D, Mathtiussi C (2008). *Bio-Inspired Artificial Intelligence: Theories, Methods, and Technologies*.

[37] Kennedy J, Eberhart RC, Shi Y (2001). *Swarm Intelligence*.

[38] Bonabeau E, Dorigo M, Theraulaz G (1999). *Swarm Intelligence: From Natural to Artificial Systems*.

[39] Engelbretch AP (2005). *Fundamentals of Computational Swarm Intelligence*.

[40] Yang XS (2010). *Nature-Inspired Metaheuristic Algorithms*.

[41] Gálvez A, Cobo A, Puig-Pey J, Iglesias A (2008). Particle swarm optimization for bézier surface reconstruction. *Lecture Notes in Computer Science*.

[42] Gálvez A, Iglesias A (2011). Efficient particle swarm optimization approach for data fitting with free knot B-splines. *CAD Computer Aided Design*.

[43] Gálvez A, Iglesias A (2012). Particle swarm optimization for non-uniform rational B-spline surface reconstruction from clouds of 3D data points. *Information Sciences*.

[44] Gálvez A, Iglesias A, Puig-Pey J (2012). Iterative two-step genetic-algorithm-based method for efficient polynomial B-spline surface reconstruction. *Information Sciences*.

[45] Yoshimoto F, Moriyama M, Harada T Automatic knot placement by a genetic algorithm for data fitting with a spline.

[46] Gálvez AG, Iglesias A, Avila A Immunological-based approach for accurate fitting of 3D noisy points with Bézier surfaces.

[47] Ülker E, Arslan A (2009). Automatic knot adjustment using an artificial immune system for B-spline curve approximation. *Information Sciences*.

[48] Zhao X, Zhang C, Yang B, Li P (2011). Adaptive knot placement using a GMM-based continuous optimization algorithm in B-spline curve approximation. *CAD Computer Aided Design*.

[49] Gálvez A, Iglesias A, Cobo A, Puig-Pey J, Espinola J (2007). Bézier curve and surface fitting of 3D point clouds through genetic algorithms, functional networks and least-squares approximation. *Lecture Notes in Computer Science*.

[50] Gálvez A, Iglesias A (2013). A new iterative mutually-coupled hybrid GA-PSO approach for curve fitting in manufacturing. *Applied Soft Computing*.

[51] Sarfraz M, Raza SA Capturing outline of fonts using genetic algorithms and splines.

[52] de Boor CA (2001). *Practical Guide to Splines*.

[53] Piegl L, Tiller W (1997). *The NURBS Book*.

[54] Yang X-S (2009). Firefly algorithms for multimodal optimization. *Lecture Notes in Computer Science*.

[55] Yang XS (2010). Firefly algorithm, stochastic test functions and design optimisation. *International Journal of Bio-Inspired Computation*.

[56] Yang XS (2011). Review of meta-heuristics and generalised evolutionary walk algorithm. *International Journal of Bio-Inspired Computation*.

[57] Yang XS, Deb S (2010). Eagle strategy using Lévy walk, and firey algorithms for stochastic optimization. *Nature Inspired Cooperative Strategies for Optimization (NICSO)*.

[58] Yang X-S, Hosseini SSS, Gandomi AH (2012). Firefly Algorithm for solving non-convex economic dispatch problems with valve loading effect. *Applied Soft Computing Journal*.

[59] Yang XS (2010). *Engineering Optimization: An Introduction with Metaheuristic Applications*.

[60] Fister I, Fister Jr I, Yang XS, Brest J (2013). Memetic self-adaptive firefly algorithm. *Swarm and Evolutionary Computation*.

[61] Gálvez A, Iglesias A (2013). Firey algorithm for polynomial Bézier surface parameterization. *Journal of Applied Mathematics*.

[62] Press WH, Teukolsky SA, Vetterling WT, Flannery BP (1992). *Numerical Recipes*.

[63] Fister I, Yang XS, Brest J, Fister I, Yang XS, Cui Z, Xiao R, Gandomi AH, Karamanoglu M (2013). Memetic self-adaptive firefly algorithm. *Swarm Intelligence and Bio-Inspired Computation: Theory and Applications*.

[64] Yang XS (2013). Multi-objective firey algorithm for continuous optimization. *Engineering with Computers*.

[65] Yang X-S, Deb S (2010). Engineering optimisation by cuckoo search. *International Journal of Mathematical Modelling and Numerical Optimisation*.

[66] Yang X-S, Deb S Cuckoo search via Lévy flights.

[67] Yang X-S (2010). A new metaheuristic Bat-inspired Algorithm. *Studies in Computational Intelligence*.

[68] Yang XS (2011). Bat algorithm for multi-objective optimisation. *International Journal of BioInspired Computation*.

[69] Yang XS, Gandomi AH (2012). Bat algorithm: a novel approach for global engineering optimization. *Engineering Computations*.

